# Hybrid *de novo* Genome Assembly of *Erwinia* sp. E602 and Bioinformatic Analysis Characterized a New Plasmid-Borne *lac* Operon Under Positive Selection

**DOI:** 10.3389/fmicb.2021.783195

**Published:** 2021-11-11

**Authors:** Yu Xia, Zhi-Yuan Wei, Rui He, Jia-Huan Li, Zhi-Xin Wang, Jun-Da Huo, Jian-Huan Chen

**Affiliations:** ^1^State Key Laboratory of Food Science and Technology, Jiangnan University, Wuxi, China; ^2^School of Food Science and Technology, Jiangnan University, Wuxi, China; ^3^Collaborative Innovation Center of Food Safety and Quality Control in Jiangsu Province, Jiangnan University, Wuxi, China; ^4^Laboratory of Genomic and Precision Medicine, Wuxi School of Medicine, Jiangnan University, Wuxi, China

**Keywords:** *Erwinia*, hybrid sequencing, genome assembly, *lac* operon, bioinformatic analysis

## Abstract

Our previous study identified a new β-galactosidase in *Erwinia* sp. E602. To further understand the lactose metabolism in this strain, *de novo* genome assembly was conducted by using a strategy combining Illumina and PacBio sequencing technology. The whole genome of *Erwinia* sp. E602 includes a 4.8 Mb chromosome and a 326 kb large plasmid. A total of 4,739 genes, including 4,543 protein-coding genes, 25 rRNAs, 82 tRNAs and 7 other ncRNAs genes were annotated. The plasmid was the largest one characterized in genus *Erwinia* by far, and it contained a number of genes and pathways responsible for lactose metabolism and regulation. Moreover, a new plasmid-borne *lac* operon that lacked a typical β-galactoside transacetylase (*lacA*) gene was identified in the strain. Phylogenetic analysis showed that the genes *lacY* and *lacZ* in the operon were under positive selection, indicating the adaptation of lactose metabolism to the environment in *Erwinia* sp. E602. Our current study demonstrated that the hybrid *de novo* genome assembly using Illumina and PacBio sequencing technologies, as well as the metabolic pathway analysis, provided a useful strategy for better understanding of the evolution of undiscovered microbial species or strains.

## Introduction

*Erwinia* is a group of the straight rod-shaped, facultative anaerobic, gram-negative bacterium of the *Erwiniaceae* family of *Enterobacteriaceae*. Most of the *Erwinia* species identified by far are pathogens, saprophytes, or epiphytes of plants. It has been reported that some *Erwinia* species ferment lactose as a carbon source. Our previous study characterized a β-galactosidase with relatively high activity at low temperature in the *Erwinia* sp. strain E602 (Xia et al., 2018).

The *lac* operons typically involve three genes encoding the enzymes that enable bacteria to utilize lactose (Diaz-Hernandez and Santillan, 2010). The gene *lacZ* encodes the β-galactosidase, an enzyme that degrades lactose into monosaccharides glucose and galactose. Similarly, *lacY* encodes a membrane-embedded transporter that helps bring lactose into cells. The gene *lacA* encodes galactoside O-acetyltransferase that catalyzes the transfer of an acetyl group from acetyl-CoA to the 6-hydroxyl of galactopyranosides, with its exact physiological function remaining unclear. In addition, the gene *lacI* encodes the *lac* repressor, which is a protein that represses the transcription of *lac* operon genes by binding to the promoter and preventing the operon from transcription when lactose is not available. In the presence of lactose, the *lac* repressor is released from the operon to allow RNA transcription (Marbach and Bettenbrock, 2012). Notably, the *lac* operon confers a competitive advantage for bacterial survival in the environments (Pinto et al., 2021). Therefore, the study of *lac* operon in the newly identified strain *Erwinia* sp. E602 may improve our understanding of the role of lactose metabolism in this genus.

High-throughput sequencing and *de novo* assembly allow obtaining the total genetic information of microbes. It is widely used to dissect the genome sequence, gene composition, and evolutionary features of novel or unknown species. Notably, long-read sequencing technologies, such as PacBio (Rhoads and Au, 2015; Zhang et al., 2021), have dramatically speeded up the understanding of microorganisms. Moreover, the whole genome sequence of microorganisms provides an important basis for post-genomics research of microorganisms, such as subsequent gene mining and functional validation. In order to further investigate the regulation mechanisms of the β-galactosidase expression in the strain *Erwinia* sp. E602, we sequenced its genome and conducted *de novo* assembly, using the hybrid Illumina and PacBio sequencing (De Maio et al., 2019). From the assembly of the *Erwinia* sp. E602 genome, we identified a large plasmid and a new plasmid-borne *lac* operon containing a *lacZ* gene encoding the β-galactosidase with low-temperature activity, as well as evidence supporting positive selection pressure of the *lac* operon.

## Results

### *De novo* Assembly and Annotation of *Erwinia* sp. E602 Genome

**FIGURE 1:**
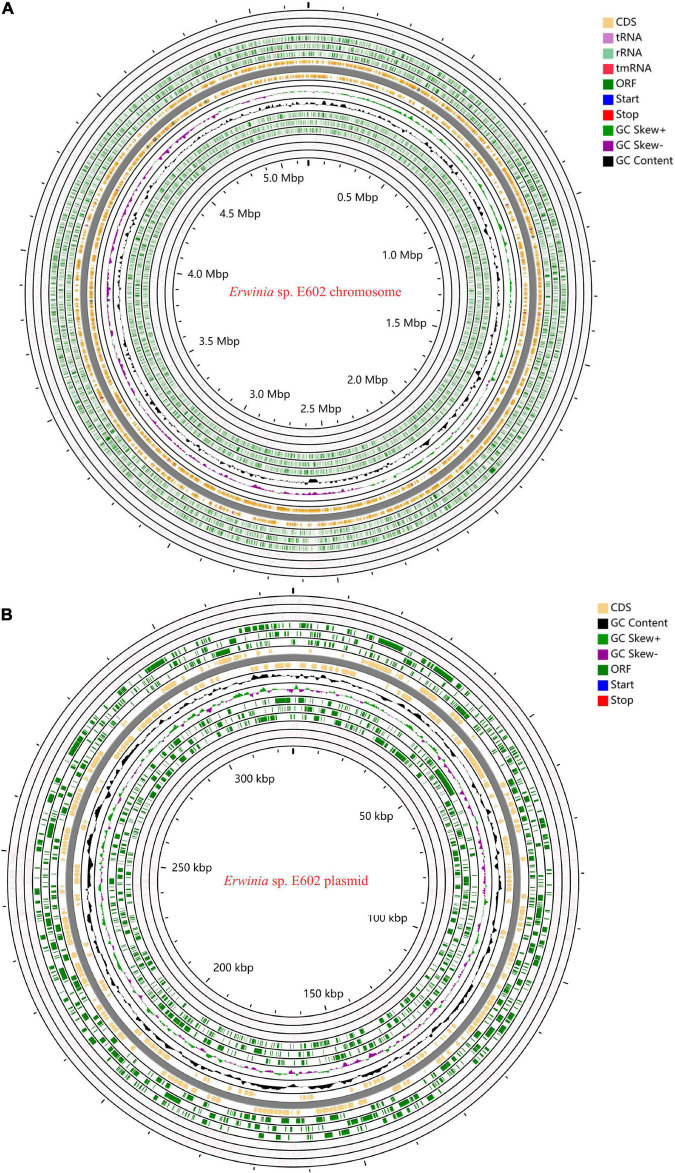
Circos plots of the annotated genome of *Erwinia* sp. E602. Two circular DNA, including the chromosome **(A)** and a large plasmid **(B)** are shown. Different colors represent CDS, rRNA, tRNA, tmRNA, Start, Stop, ORF, GC content, GC Skew+, and GC skew- respectively.

As summarized in Supplementary Tables 1–3, the Illumina and PacBio reads were used to conduct *de novo* genome assembly. After hybrid *de novo* assembly using SPAdes, two circular scaffolds were obtained, including a chromosome sequence of 4,842,717 bp with a GC content of 58.91% (GenBank: CP046582.1) and a large plasmid sequence of 325,969 bp (GenBank: CP046581.1). A total of 4,739 genes, including 4,543 protein-coding, 25 rRNAs, 82 tRNAs, and 7 other ncRNAs genes were annotated as shown in Table 1 and Figures 1A,B. Among these genes, 4,472 genes including 4,296 protein-coding, 25 rRNAs, 82 tRNAs, and 7 other ncRNAs genes were located on the chromosome, while 277 protein-coding genes were located on the large plasmid.

**TABLE 1:** Summary of *Erwinia* sp. E602 genome annotation.

**Type**	**Chromosome**	**Plasmid**
RefSeq	NZ_CP046582.1	NZ_CP046581.1
INSDC	CP046582.1	CP046581.1
Size (Mb)	4.84	0.33
GC%	58.9	59.0
Protein	4,296	277
rRNA	25	–
tRNA	82	–
Other RNA	7	–
Gene	4,472	82
Pseudogene	62	5

EggNOGv5.0 was then used to predict the functions of these genes. The KEGG pathway enrichment analysis was also performed (Supplementary Material 1). Pathways with a number of genes greater than 5 were shown, and finally, 122 enriched pathways were obtained. The most-enriched pathways (gene counts) included biosynthesis of secondary metabolites (357), microbial metabolism in diverse environments (260), ABC transporters (204), biosynthesis of cofactors (156), biosynthesis of amino acids (132), two-component systems (115), carbon metabolism (98), purine metabolism (71), quorum sensing (63), pyrimidine metabolism (51), ribosome (51), cysteine and methionine metabolism (50), bacterial secretion system (48), and amino sugar and nucleotide sugar metabolism (47). By dbCAN2 database annotation (Zhang et al., 2018), 593 carbohydrases were found on the chromosome and 51 on the plasmid (Supplementary Material 1). Through Resfams database annotation (Gibson et al., 2015), we found 211 resistance genes on the chromosome and 21 resistance genes on the plasmid, respectively (Supplementary Material 1).

### New Plasmid-Borne *lac* Operon in *Erwinia* sp. E602

**FIGURE 2:**
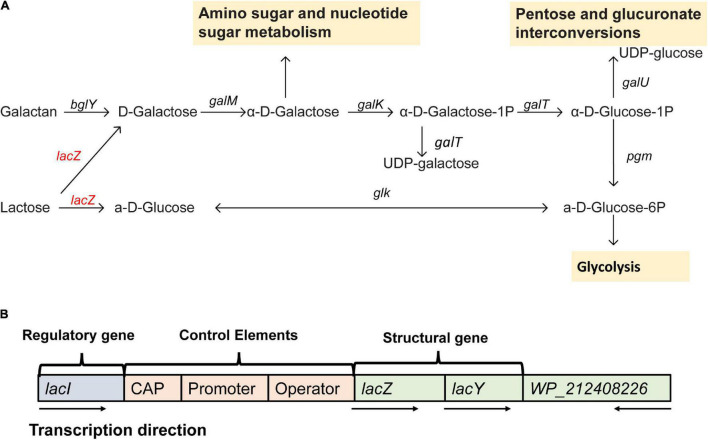
A new plasmid-borne *lac* operon characterized in *Erwinia* sp. E602. **(A)** Lactose-galactose metabolic pathway. Gene names are above the arrow, and the yellow box represents the downstream metabolic pathway. The red color indicates that the gene is located on the plasmid and black means that the gene is located on the chromosome. **(B)** A structure diagram of the new *lac* operon and its downstream *lacI*-like gene encoding the WP_212408226 protein. The arrows indicate the transcription directions of the genes.

From the gene annotation results of *de novo* assembly, *Erwinia* sp. E602 was found to contain a number of genes and pathways for lactose metabolism and regulation (Figure 2A). A list of lactose/galactose-related functional genes were shown in Table 2.

**TABLE 2:** Lactose/galactose related functional genes annotated in *Erwinia* sp. E602 genome.

**Function**	**Counts**	**Gene name**
*lac* operon repressor	11	*lacI, AscG, galS, scrR, cytR, aglR, gntR, aglR, etc.*
Beta-galactosidase	3	*lacZ, lacZ3, bglY*
Lactose/galactose transport	9	*lacY, lacY-2, mglA-1, mglA, celC, ulaB, ptxB, bglE*
Galactose operon repressor	1	*galR*

**FIGURE 3:**
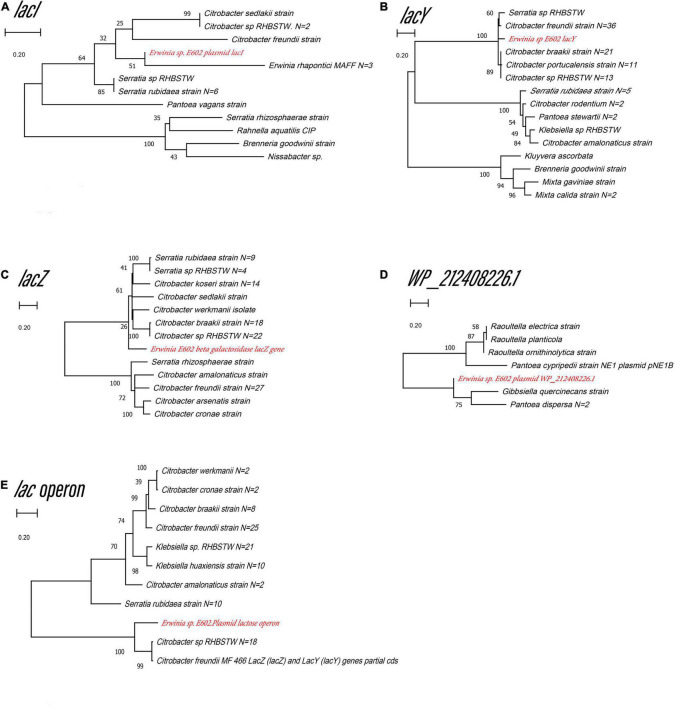
Unrooted maximum likelihood phylogeny of the four plasmid-borne *lac* operon and *lacI*-like (WP_212408226) genes. Phylogeny trees of the genes *lacI* **(A)**, *lacY* **(B**), *lacZ* **(C)**, *lacI*-like (WP_212408226) **(D)**, *lac* operon **(E)** based on gene sequences. These trees were obtained using software MEGA7 by the Neighbor-Joining method. Numbers at branch nodes indicate bootstrap values of 1,000 trials (only bootstrap values above 50% were shown). Bar = 0.20 substitutions per nucleotide site.

Our previous study characterized a new β-galactosidase that exhibited activity at low temperatures, indicating its potential unique biological function (Xia et al., 2018). Intriguingly, the *de novo* assembly results showed that the exact *lacZ* gene encoding the new β-galactosidase was not located on the chromosome of *Erwinia* sp. E602. Instead, further analysis of the sequence context surrounding the *lacZ* gene found that the gene was a component of a new *lac* operon on the large plasmid. The *lac* operons are known to be composed of regulatory genes, manipulated regions, and structural genes (Diaz-Hernandez and Santillan, 2010). The newly identified *lac* operon in the current study contained *lacI, lacZ, lacY*, as well as a regulatory element-CAP and a promoter, between the location of *lacI* and *lacZ* (Figure 2B). BLAST results of the plasmid-borne *lac* operon showed that it shares low sequence similarity with other sequences in other *Erwinia* strains. These results are included in Supplementary Materials. Moreover, this newly discovered *lac* operon, compared with the classical ones, lacked a *lacA* gene but had a reversed *lacI*-like gene encoding protein WP_212408226. Further comparison of the related genes revealed that although other *lacZ*, *lacI* and *lacY* paralogs were also observed in the genome of *Erwinia* sp. E602, they did not form a canonical *lac* operon due to the lack of essential structure. Moreover, the *lacI* and *lacY* genes of the plasmid-borne *lac* operon share low similarity with their homologs in the genomes of *Erwinia* species (Table 3), suggesting that this *lac* operon was distinct from canonical ones. In addition, the NCBI BLAST search using the nucleic acid database was performed to identify potentially similar operon-related genes, and the results showed that the genes in this operon were more similar to those in genus *Citrobacter* (Figure 3). Likewise, the *lacZ* gene of the plasmid-borne *lac* operon shared a relatively low identity with its paralogs in the other *Erwinia* species (Table 4). Instead, its *lacZ* and *lacY* had high similarities with their homologs in *Citrobacter* species. The genes of *lacI* and *lacI-*like *protein* (WP_212408226) were compared using the NCBI nucleic acid database, and no sequences with similarities greater than 80% were found. In addition, no *lacA* was found either on the chromosome of Erwinia sp. E602, or on its large plasmid.

**TABLE 3:** Sequence similarity of *lac* operon related genes.

**Title**	**Query coverage**	***E-*value**	**Per. ident**
*plasmid_238-lacI vs. genome_2601-lacI*	41%	2.00 E-38	72.04%
*plasmid_238-lacI vs*. *plasmid_241_wp*	4%	7.00 E-06	89.29%
*genome_2601–lacI vs*. *plasmid_241_wp*	3%	0.004	100.00%
*plasmid_240-lacY vs*. *genome_2604-bglY*		No sig	
*plasmid_240-lacY vs. genome_3788-lacY*	94%	5.00 E-161	70.88%
*genome_2604-bglY vs. genome_3788-lacY*		No sig	
*plasmid_239-lacZ vs. plasmid_250-lacZ3*		No sig	
*plasmid_239-lacZ vs. plasmid_240-lacY*	0	0.039	100.00%

**TABLE 4:** Identity of plasmid-borne *LacZ* in *Erwinia* sp. E602 with its paralogs in other *Erwinia* species.

***Erwinia* strains**	**Chromosome/Plasmid**	**Counts**	**GI (Identity%)**
*Erwinia amylovora* CFBP1430	Chromosome	3	GI:490258078 (13%); GI:490258079 (13%); GI:490258942 (61%)
*Erwinia billingiae* Eb661	Chromosome	2	GI:502965951 (16%); GI:502966002 (65%)
*Erwinia gerundensis* E_g_EM595	Chromosome	2	GI:1055842378 (64%); GI:1055871078 (16%)
*Erwinia gerundensis* E_g_EM595	Plasmid pEM01	1	GI:1055874842 (59%); GI:1055874917 (11%)
*Erwinia* sp. J780	Chromosome	2	GI:1783198844 (15%); GI:1783198856 (12%); GI:1783199144 (64%)
*Erwinia* sp. QL-Z3	Chromosome	2	GI:1606668623 (16%); GI:1828892330 (65%)
*Erwinia tasmaniensis* Et1/99	Chromosome	1	GI:501410713 (62%)

### Selective Pressure in Genes in the Newly Identified *lac* Operon

To better understand whether genes in the plasmid-borne *lac* operon are subject to natural selection during adaptation to the environment, positive selection sites were identified using branch-site models (model A vs. model A null) and site models (M1a vs. M2a; M7 vs. M8) implemented in Phylogenetic Analysis by Maximum Likelihood (PAML) (20). Due to fewer sequences similar to genes *lacI* and the *lacI-*like protein (WP_212408226) in other bacteria strains, we chose the top 100 sequences ranked by identity with *lacY* or *lacZ* to be included in the two datasets separately for selection pressure analysis. The results of the branch-site model comparison showed that the gene *lacY* had a site at codon 187 under positive selection (Table 5). Surprisingly, there are multiple sites under strong positive selection in the gene *lacZ*, indicating the strong positive selection pressure in it (Table 6).

**TABLE 5:** Bayes Empirical Bayes analysis of positively selected sites identified in *lacY* of *Erwinia* sp. E602 with the branch-site model A.

**Model**	**Codon**	**Amino acid**	**Posterior probability**
Branch-site model	75	T	0.663
	147	G	0.564
	187	G	0.956*
	191	T	0.748
	207	S	0.801

*Posterior probability from BEB analysis > 95%; ratio for foreground branch (ω > 1).

**TABLE 6:** Positively selected sites in *lacZ* of *Erwinia* sp. E602 identified with site model M2a using Bayes Empirical Bayes analysis.

**Codon**	**Amino acid**	**Probability**	**Post mean**
29	A	0.91	1.767
34	R	0.991**	1.873
37	I	0.901	1.755
38	T	0.960*	1.833
39	L	0.959*	1.83
114	T	0.936	1.801
115	G	1.000**	1.884
140	S	0.987*	1.867
146	V	0.9	1.755
151	A	0.974*	1.851
159	S	0.974*	1.85
386	S	0.995**	1.877
412	N	0.901	1.756
421	R	0.979*	1.857
425	P	0.998**	1.882
426	A	0.926	1.788
427	T	0.955*	1.825
429	R	0.995**	1.878

*Posterior probability of BEB analysis > 95%; **, posterior probability of BEB analysis > 99%. BEB, Bayes Empirical Bayes.

## Discussion

By using a strategy combining Illumina and PacBio sequencing technology, the *de novo* assembly of the whole genome of *Erwinia* sp. E602 was performed. Moreover, our study characterized the largest plasmid in the genus *Erwinia* by far, and reported a new plasmid-borne *lac* operon.

From the assembly, a large plasmid with a length of 325,969 bp and a total of 297 genes was characterized. Before our current study, 6 plasmids had been reported in the genus *Erwinia*, among which the largest plasmid was CP037949 derived from *Erwinia* sp. QL-Z3. That plasmid was 149,889 bp in length and encoded a total of 124 genes.^1^ Therefore, the plasmid characterized in our current *de novo* genome assembly of *Erwinia* sp. E602 is the largest plasmid found in the genus *Erwinia* by far. In general, the genes in plasmids might provide microorganisms with potential genetic advantages. Yet the specific role of the large plasmid in *Erwinia* sp. E602 still remains unclear.

The *lac* operon is a hallmark gene of the regulatory circuit for bacteria to regulate metabolism according to nutrient conditions in the environment (Leonard et al., 2015; Malakar, 2015; Karkare et al., 2021; Pinto et al., 2021). Through genome function annotation, a large number of genes related to lactose metabolism were found in *Erwinia* sp. E602, a cold-adapted strain. In particular, a new plasmid-borne *lac* operon was characterized in this study. The *lac* operon shared low similarity with homologous genes in *Erwinia* species. It lacked a typical *lacA*. Instead, the position of β-galactoside transacetylase was replaced by a *lacI*-like gene encoding uncharacterized function. The gene *lacZ* in the newly identified *lac* operon encodes a β-galactosidase with the characteristics of low-temperature adaptation (Xia et al., 2018), which might help the organism survive in extreme environments. Similarly, identification and isolation of a 127-kb large plasmid in a rat *E. coli* isolate (EC93), containing the *cdiI* gene, would show higher toxic potency, thus helping it to exert a competitive advantage (Waneskog et al., 2021). Plasmids are mobile parts of bacterial genomes that carry genes that benefit the survival of the organism and confer selective advantages such as antibiotic resistance, thus helping bacterial communities to quickly adapt to the environment (Bogomazova et al., 2020). Similarly, the large plasmid we found in *Erwinia* sp. E602 also contains a group of I toxin-antitoxin modules, mainly including *vagC, vapC, relE, stbD, yefM*, and other related genes.

The plasmid-borne *lac* operon might enable *Erwinia* sp. E602 to utilize substrates from environmental resources for lactose metabolism, thereby gaining an adaptive advantage over competitors. Similar functions have been found in *E. coli* (Harwani, 2014). Nevertheless, our analysis showed that the *lac* operon in *Erwinia* sp. E602 had low sequence similarity to that of *E. coli* and was evolutionarily distant from its *E. coli* counterparts. Likewise, other studies reported the *lac* operons in specific strains (Vaughan et al., 1998; Bidart et al., 2018), which also suggested that the *lac* operon could vary in different habitats. Furthermore, the *lacZ* and *lacY* genes on the *lac* operon in *Erwinia* sp. E602 were under positive selection, which might benefit the bacterium in terms of survival or competition (Bundalovic-Torma et al., 2020). Meanwhile, the strain *Erwinia* sp. E602 was isolated from the high latitudes of northeast China and the enzyme encoded by the plasmid-borne *lacZ* gene exhibited good activity at low temperatures. These findings might be in line with the possible adaptation of this *Erwinia* strain to the cold environment.

The plasmid-borne *lac* operon lacked the *lacA* gene in *Erwinia* sp. E602. It has been reported that the transacetylase encoded by the *lacA* gene is not an essential element for lactose catabolism (Lagesen et al., 2007). In contrast, a *lacI*-like gene was found in the opposite strand downstream of the *lac* operon in *Erwinia* sp. E602. It remains to be elucidated whether the *lacI*-like gene contributes to the regulation of the *lac* operon.

In the current study, by combining Illumina and PacBio sequencing technologies, a hybrid *de novo* assembly was performed to obtain a more accurate and complete map of the genome of *Erwinia* sp. E602, providing a reference basis for subsequent transcriptome studies. Furthermore, a new plasmid-borne *lac* operon was characterized with evidence supporting evolution and adaptation to the environment of the genus *Erwinia*. The specific role of the large plasmid on *Erwinia* sp. E602 needs further studies.

## Materials and Methods

### Bacteria Culture and DNA Extraction

Bacterial culture and DNA extraction of *Erwinia* sp. E602 were performed as described in our previous report (Xia et al., 2018). The genomic DNA was used for subsequent Illumina and PacBio sequencing.

### Illumina Sequencing and Data Processing

The library for short read sequencing was prepared from 1 μg *Erwinia* sp. E602 genomic DNA using NEBNext Ultra DNA Library Prep Kit (NEB, Ipswich, Massachusetts) according to the manufacturer’s instructions, assessed using a Qubit^®^ 2.0 Fluorometer (Invitrogen, Waltham, Massachusetts) and an Agilent 2100 Bioanalyzer (Agilent, Santa Clara, California) for quality control (QC), and sequenced on the Hi-Seq 2500 platform (Illumina, San Diego, California) using a 100 bp paired-end configure. All adapter sequences and low-quality bases contained in the reads were filtered and removed using Trimmomatic (version 0.31), to keep only reads with sequencing read length more than 90 nucleotides (nt), and ensure the proportion of base quality greater than 20 (Q20) was not less than 90%.

### PacBio Sequencing and Data Processing

The library for single-molecule long read sequencing was also prepared from 5 μg genomic DNA using SMRTbell DNA Template Prep Kit 2.0 (Pacific Biosciences, Menlo Park, California) according to the manufacturer’s instructions, assessed using a Qubit^®^ 2.0 Fluorometer and an Agilent 2100 Bioanalyzer for quality control (QC), and sequenced on the PacBio RS System. Raw data was filtered to obtain clean data with a cutoff of minimum polymerase read quality 0.75 and minimum polymerase read length 3,500 bp.

### Hybrid Assembly of Illumina and PacBio Sequencing Data

The filtered Illumina reads and PacBio subreads were then used to conduct the hybrid assembly using SPAdes software (v3.5.0) (Bankevich et al., 2012). PacBio subreads were provided to SPAdes using the –PacBio option for gap closure and repeat resolution.

### Gene Prediction

The software Prokka (Seemann, 2014) (v 1.14.6) was used to predict genes in the assembly results, Carbohydrate enzymes were annotated using dbCAN2 (Zhang et al., 2018). Resistance genes were annotated using the Resfams database (Gibson et al., 2015). The prediction of rRNA was performed using rnammer1.2 (Lagesen et al., 2007). The prediction of tRNA was performed by the software tRNAscan-SE-1.23 (Chan and Lowe, 2019). The sRNA annotation was obtained by the software infernal-1.1rc4 (Nawrocki and Eddy, 2013). The CGView server (Stothard and Wishart, 2005) was used as an interactive comparison genomics tool to draw the circos plots.

### Functional Annotation and Pathway Enrichment Analysis

EggNOG v5.0 (Huerta-Cepas et al., 2019) were used for the functionally annotation of genes. Pathway enrichment analysis was performed using Kyoto Encyclopedia of Genes and Genomes (KEGG) pathway and KEGG Orthology (KO) databases.^2^

### Prediction of *lac* Operon

The prediction of *lac* operon was conducted using Operon-mapper (Taboada et al., 2018).

### Phylogenetic Analysis

Phylogenetic analysis was performed using software MEGA7 (Kumar et al., 2016) using the Neighbor-Joining method. The 1,000 replicates of bootstrap test were used to evaluate the clustering of taxa at branch nodes.

### Select Pressure Assessment

In order to analyze the selection pressure of the related genes, PAML (v4.1) was used to identify sites under positive selection in the genes with site and branch-site models (Yang, 2007). For the branch-site models, *Erwinia*. sp. E602 was selected as the foreground branch of the branch-site model, and other bacteria were used as the background branch.

## Data Availability Statement

The datasets presented in this study can be found in online repositories. The names of the repository/repositories and accession number(s) can be found in the article/Supplementary Material.

## Author Contributions

YX and J-HC conceived the project, planned the experiments, and participated in the results discussions. Z-YW performed the bioinformatic analysis and improved the data analysis pipelines. RH participated in the DNA extraction from bacteria. J-HL, Z-XW, and J-DH participated in the data analysis. All authors contributed to the final manuscript.

## Conflict of Interest

The authors declare that the research was conducted in the absence of any commercial or financial relationships that could be construed as a potential conflict of interest.

## Publisher’s Note

All claims expressed in this article are solely those of the authors and do not necessarily represent those of their affiliated organizations, or those of the publisher, the editors and the reviewers. Any product that may be evaluated in this article, or claim that may be made by its manufacturer, is not guaranteed or endorsed by the publisher.

## Footnotes

1
https://www.ncbi.nlm.nih.gov/genome/browse/#!/plasmids/13524/

2
https://www.genome.jp/kegg/mapper/search.html
